# Aptamer functionalized nucleic acid nano drug for targeted synergistic therapy for colon cancer

**DOI:** 10.1186/s12951-023-01941-z

**Published:** 2023-06-07

**Authors:** Liye Zhu, Jieyu Yuhan, Hao Yu, Boyang Zhang, Longjiao Zhu, Xiaoyun He, Kunlun Huang, Wentao Xu

**Affiliations:** 1 Food Laboratory of Zhongyuan, Key Laboratory of Precision Nutrition and Food Quality, Department of Nutrition and Health, China Agricultural University, No. 17 Qinghua Donglu, Beijing, 100083 China; 2 College of Food Science and Nutritional Engineering, China Agricultural University, Beijing, 100083 China; 3 College of Veterinary Medicine, China Agricultural University, Beijing, 100094 China

**Keywords:** Anti-mir-21, AS1411 aptamer, Dox, Rolling circle transcription, colon cancer, Nanotherapeutics

## Abstract

Due to its complicated pathophysiology, propensity for metastasis, and poor prognosis, colon cancer is challenging to treat and must be managed with a combination of therapy. Using rolling circle transcription (RCT), this work created a nanosponge therapeutic medication system (AS1411@antimiR-21@Dox). Using the AS1411 aptamer, this approach accomplished targeted delivery to cancer cells. Furthermore, analysis of cell viability, cell apoptosis, cell cycle arrest, reactive oxygen species (ROS) content, and mitochondrial membrane potential (MMP) levels revealed that functional nucleic acid nanosponge drug (FND) can kill cancer cells. Moreover, transcriptomics uncovered a putative mechanism for the FND anti-tumor effect. These pathways, which included mitotic metaphase and anaphase as well as the SMAC-mediated dissociation of the IAP: caspase complexes, were principally linked to the cell cycle and cell death. In conclusion, by triggering cell cycle arrest and apoptosis, the nano-synergistic therapeutic system allowed for the intelligent and effective targeted administration of RNA and chemotherapeutic medicines for colon cancer treatment. The system allowed for payload efficiency while being customizable, targeted, reliable, stable, and affordable.

**Supplementary Information:**

The online version contains supplementary material available at 10.1186/s12951-023-01941-z.

## Introduction

The third most frequent cancer in the world and the fourth most common cause of cancer-related mortality is colon cancer [1]. It is difficult to treat due to its complex pathogenesis, susceptibility to metastasis, and poor prognosis [2, 3]. Currently, chemotherapy, targeted therapy, and surgical excision of the original tumor are the mainstays of advanced colon cancer treatment. Chemotherapy is typically successful in slowing the growth of tumor cells and preventing the spread of metastatic disease [4]. Dox, a common chemotherapy drug, successfully treats advanced colon cancer by preventing DNA replication and causing oxidative stress, which results in DNA damage and cell death [5]. However, because it avoids dangerous side effects including nausea, vomiting, and cardiotoxicity, synergistic therapy is currently a well-liked cancer treatment [6, 7].

Chemotherapy and gene therapy used in conjunction is a successful cancer treatment method. Numerous researches have looked into the relationship between abnormal miRNA expression and the onset and development of colon cancer [8, 9]. Because miRNAs have diagnostic and prognostic significance for those who have the disease, they can be exploited as potential novel targets for colon cancer therapy [10]. Studies have found a correlation between high levels of miR-21 expression in tumors and a poor prognosis and chemotherapeutic response in people with colon cancer [11, 12]. miR-21 can participate in tumor pathogenesis and development, including cell proliferation, migration, invasion, metastasis, and apoptosis, by targeting PTEN, PDCD4, TIMP3, and RHOB, or signaling pathways, such as RAS/MEK/ERK, PTEN/PI-3 K/AKT, and Wnt/β-catenin [13, 14]. Li et al. used plasmids encoding miR-21 inhibitors to transfect the DLD-1 cell line, which had low miR-21 expression, and the SW480 cell line, which had high miR-21 expression. They discovered that deletion of the miR-21 gene prevented SW480 cells from proliferating, migrating, and invading while miR-21 gene overexpression encouraged these behaviors in DLD-1 cells [15]. In vivo results indicated that miR-21 overexpression promoted tumor growth in BALB/c nude mice [16]. This showed how crucially miR-21 functions at the molecular level in colon cancer, pointing to it as a possible target for colon cancer treatment. Additionally, miRNA research has moved from the lab to the clinic. Over 15 miRNA drugs that are in various stages of clinical development are listed on Clinicaltrials.gov, Clinicaltrialsregister.eu, and research.cicc.com. Additionally, over 11 miRNA antisense oligonucleotides are readily available.

Targeted drug delivery can guarantee efficient cancer treatment with minimal hazardous side effects. Aptamers, which are typically short nucleic acid sequences of 20–80 nucleotides (ssDNA or ssRNA), fold into stable three-dimensional structures and bind to targets—also known as “chemoantibodies”—with great affinity and specificity [17]. Aptamers have special benefits such high specificity, quick tissue and organ penetration, simplicity of chemical modification, minimal toxicity, and low immunogenicity [18]. The AS1411 aptamer is made up of 26 bases and has a high affinity for nucleolin, a protein that is widely expressed on the cell membrane of cancer cells [19, 20]. It inhibits nuclear factor NF-B and destabilizes anti-apoptotic Bcl-2 protein mRNA by binding to nucleolin proteins in the cytoplasm via nucleolin-mediated internalization, which results in the death of tumor cells [21, 22]. According to studies, the AS1411 aptamer could be utilized to deliver siRNA and treat melanoma [23]. The AS1411 aptamer can effectively target cancer cells as a result. This work makes use of the aptamer’s targeting and functionality for colon cancer treatment.

**Fig. 1:**
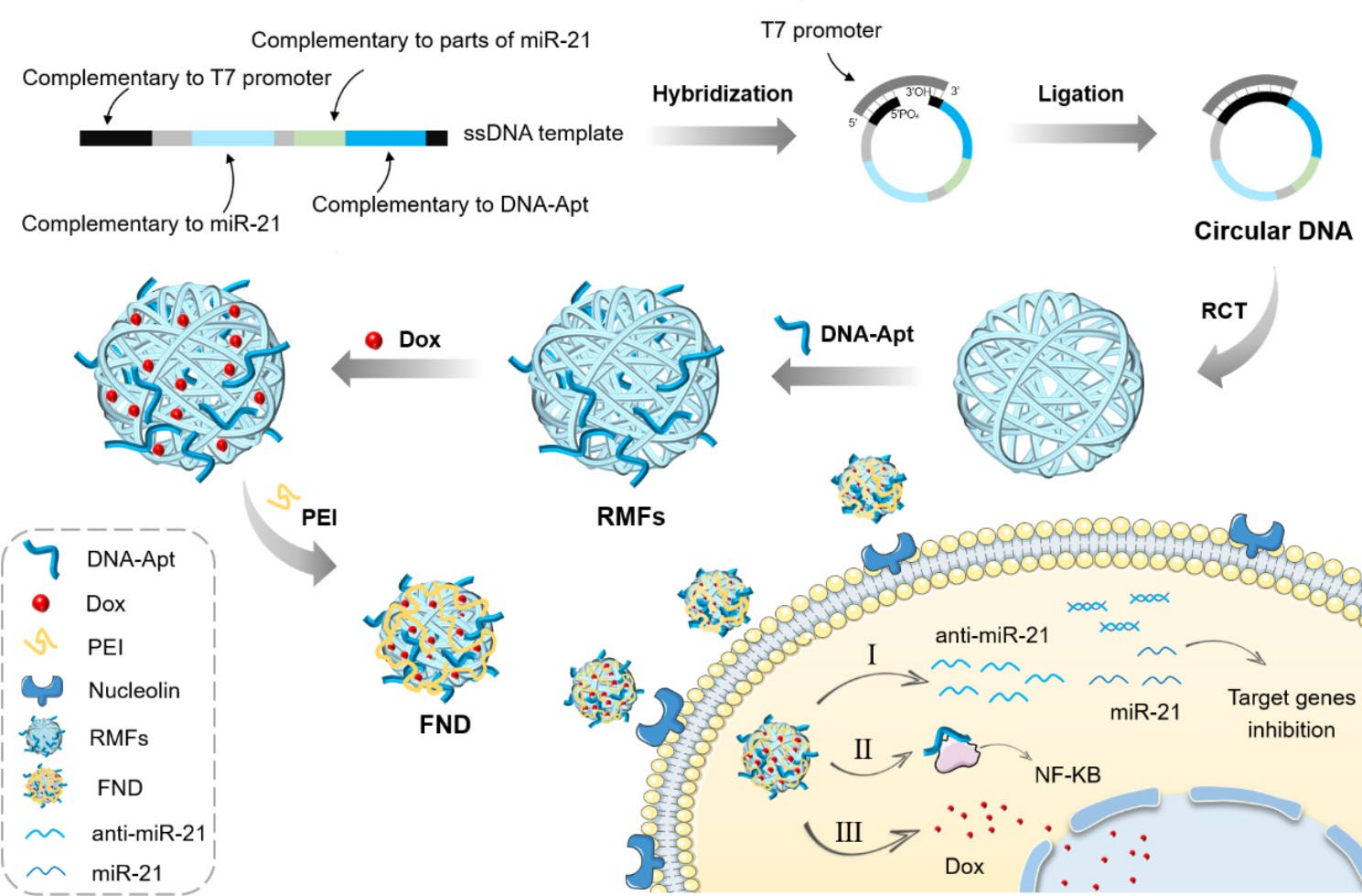
Schematic diagram of the FND preparation principle and application

For the treatment of colon cancer, an aptamer-functionalized nucleic acid nanosponge drug (FND) system (AS1411@ antimiR-21@ Dox) has been developed. (Fig. 1). In order to create anti-miR-21 nanospheres with a lot of AS1411 aptamer binding sites and space for high-abundance loading, the system uses rolling circle transcription (RCT). RCT is transcribed from circular DNA to produce a number of extended RNA molecules, which then condense into spherical particles to form RNA sponge balls [24]. Compared to single-stranded RNA, the RNA molecules in these sponges are more stable. In order to create the nanosponge drug (NSD) system, Dox is then loaded into the RNA sponge balls thanks to its flattened aromatic ring, which may be inserted between the -G-C-base pairs [25]. As a result, the FND, antimiR-21, and AS1411 aptamer are developed via RCT. In conclusion, this nanosponge drug system allows for the combined use of chemical and nucleic acid medications to treat colon cancer since it is customizable, controlled, targeted, affordable, and able to carry a large payload.

## Results and discussion

### Preparation and characterization of the RCT amplicon

**Fig. 2:**
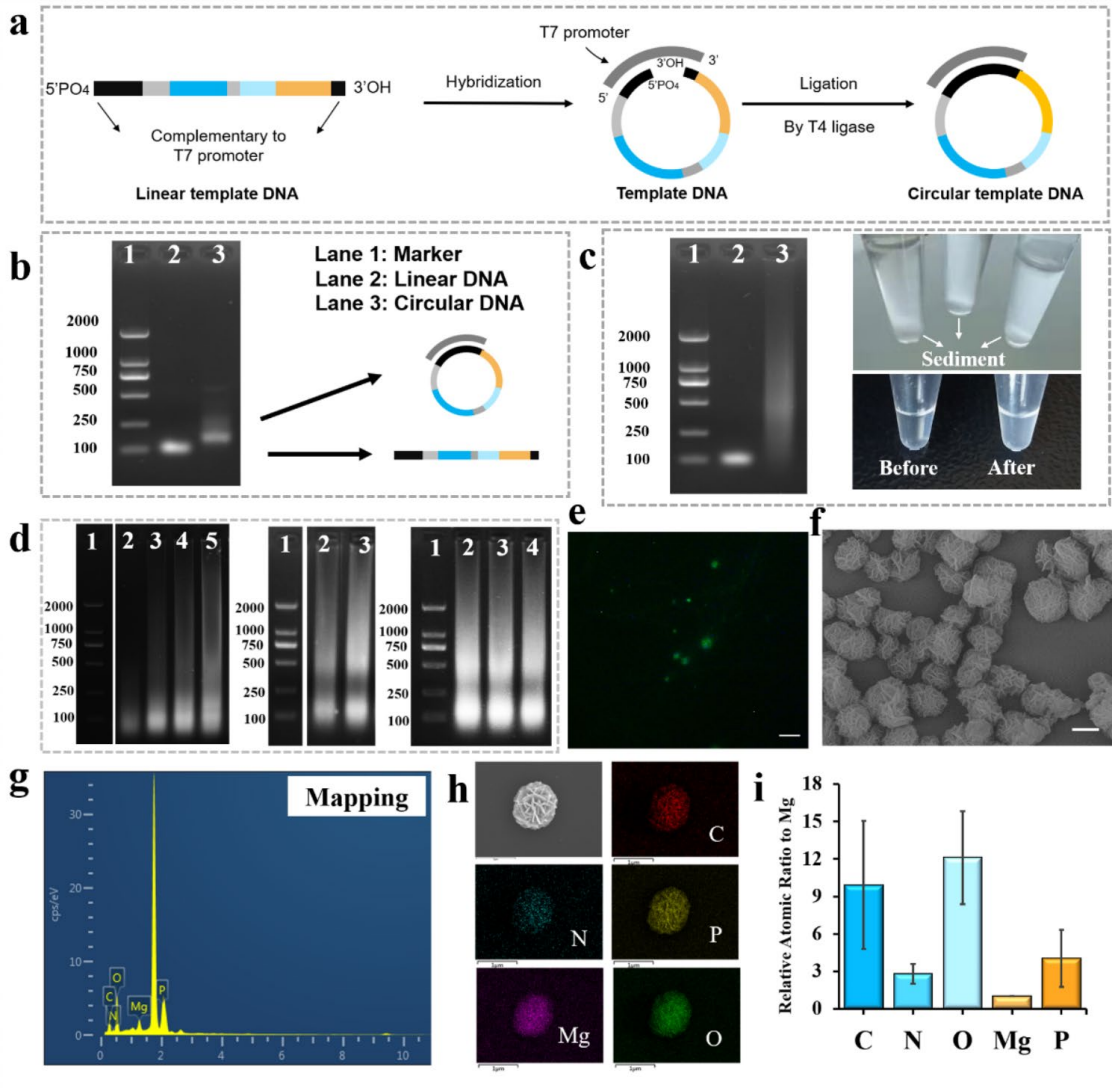
(**a**) Schematic diagram of the construction of the circular template used for RCT. (**b**) 2% agarose gel image. Lane 1: 2 K bp DNA Ladder; lane 2: linear DNA template; lane 3: circular DNA template. (**c**) Agarose gel to verify the feasibility of RCT (lane 1: 2 K bp DNA ladder; lane 2: circular DNA template; lane 3: RCT-based product) and illustration. (**d**) Agarose gel electrophoresis images of RCT products at different template concentrations (lane 1: 2 K bp DNA ladder; lane 2: 0.25 µM; lane 3: 0.5 µM; lane 4: 1 µM; lane 5: 2.5 µM), T7 RNA polymerase concentration (lane 1: 2 K bp DNA ladder; lane 2: 2.5 U/µL; lane 3: 5 U/µL), and DTT concentration (lane 1: 2k bp DNA ladder; lane 2: 1 mM; lane 3: 2.5 mM, lane 4: 5 mM). (**e**) SYBR Gold staining to verify the feasibility of RCT (scale bar: 20 μm). (**f**) SEM to verify the feasibility of RCT (scale bar: 1 μm). (**g**) Element composition diagram of RMFs. (**h**) EDS images of C, N, Mg, O, P (scale bar: 1 μm). (**i**) Content of elements C, N, O and P (normalized by reference to Mg)

As shown in Fig. 2a, The RCT reaction depended on creating a circular DNA template. A DNA template-promoter complex was created by annealing a single-stranded DNA template that had been phosphorylated at the 5’ end with a promoter. This complex could then be cycled in the presence of T4 DNA ligase. Agar gel electrophoresis was used to determine the characteristics of the circular products based on the size and structural variations of the molecules. In Fig. 2b, lanes 2 and 3 exhibit a single-stranded DNA template and a complex that has undergone annealing and T4 DNA ligase treatment, respectively. Figure 2b showed that lane 3 migrated at a slower rate, which was consistent with the results obtained by previous studies [26], illustrating the formation of the circular DNA template. To further ensure that the majority of the template could hybridize with the promoter to form the loop, the ratio of the template concentration to the promoter concentration in this study was set at 1:1.2 for the RCT based on references in the existing literature and preliminary experimental optimization.

With the addition of DNA-Apt annealing, the subsequent RCT reaction was carried out under the optimum conditions mentioned above. Prior to the reaction, the system was clear and transparent, but after the reaction was finished, it appeared cloudy with a discernible white precipitate at the bottom of the EP tube (Fig. 2c). The products underwent 2% agarose gel electrophoresis after being centrifuged-washed. The nucleic acid product from Lane 3 had a large molecular weight, demonstrating the effectiveness of the rolling loop transcription procedure (Fig. 2c).

The amounts of the template, T7 RNA polymerase, and DTT were adjusted, and agarose gel electrophoresis was used to describe them in order to produce a better rolling loop transcription system. Large molecular weight nucleic acid chains were produced as the concentration of the circular template rose, as evidenced by the appearance of visible bands with slow migration rates that become brighter (Fig. 2d). The concentration of the cyclic template utilized in the following experiments was chosen at 1 M after taking the yield and synthesis cost into account. When the T7 RNA polymerase concentration was optimized (Fig. 2d), Lane 3 became brighter, indicating a rise from 2.5 U/L to 5 U/L. It was decided to optimize the T7 RNA polymerase concentration at 5 U/L. The bands in lanes 2 to 4 with a different DTT concentration did not show any discernible variations, showing that the DTT concentration had a negligible impact on the product quantity (Fig. 2d). RCT is a time-dependent reaction that affects RCT yield and product particle size, according to reports [24]. Dynamic light scattering was used in this investigation to describe the products at 8 h, 16 h, 24 h, and 32 h (Table S1). The longer the reaction period, the larger the product and the lower the potential, suggesting that the nucleic acids were created gradually. The reaction was finished at 24, as evidenced by the fact that the product size and potential did not vary appreciably during 36 h as compared to 24 h. As a result, the optimal reaction time for RCT was 24 h.

For the purpose of initially confirming the presence of nucleic acid products, the precipitate was stained with SYBR Gold. The results revealed a large number of green fluorescent spots (Fig. 2e), demonstrating that the precipitate was created by the co-assembly of nucleic acids and did not contain or consist exclusively of magnesium pyrophosphate. Additionally, the products underwent field emission scanning electron microscopy characterization. Rolling ring-based transcriptional micron flowers (RMFs) are spherical, flower-like structures with micron dimensions that showed folded lamellae on their surfaces. The existence of C, N, O, Mg, and P was confirmed by scanning electron microscopy (SEM) and energy dispersive spectroscopy (EDS) elemental mapping (Fig. 2g and h). Additionally, the volume was estimated (Fig. 2i). Magnesium pyrophosphate (Mg_2_P_2_O_7_•3.5H_2_O) crystals are formed during RCT due to the Mg^2+^ reaction in the buffer with the pyrophosphate anion (PPi), a by-product of nucleic acid polymerization [27, 28]. The energy spectrum results supported earlier research on RNA polymerization using cyclic DNA templates [26, 29], demonstrating the viability of the RCT method.

### Preparation and characterization of FND

**Fig. 3:**
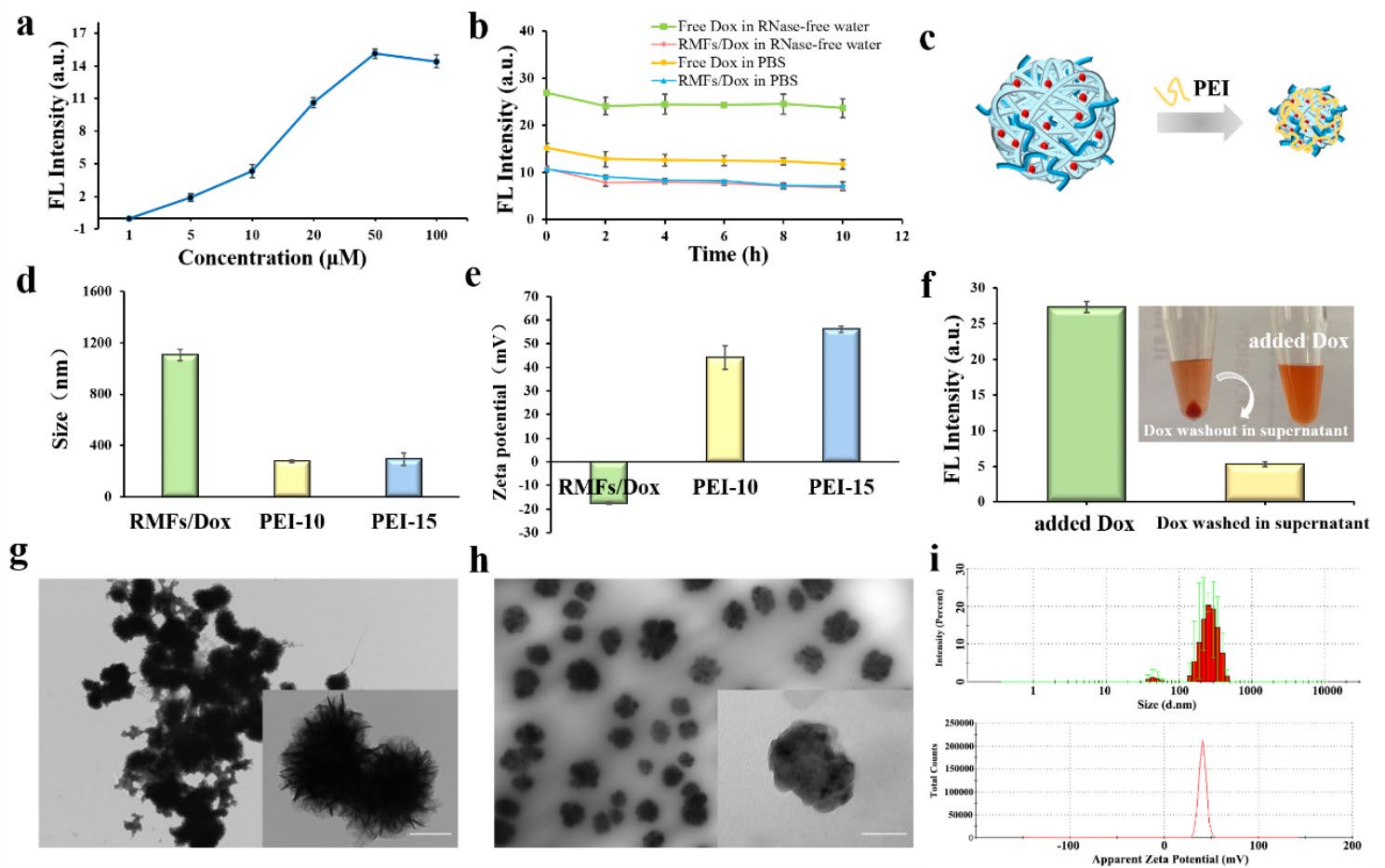
(**a**) Load optimization of RMFs for Dox. (**b**) Stability of RMFs loading Dox. (**c**) Schematic of PEI compression RMFs. (**d**) Size before/after PEI concentration optimization. (**e**) Zeta potential before/after PEI concentration optimization. (**f**) Quantification of RMFs wrapped around Dox. (**g**) TEM image of RMFs (scale bar: 500 nm). (**h**) TEM image of FND (scale bar: 100 nm). (**i**) Particle size and potential characterization of FND.

RMFs were used for Dox co-delivery to load Dox. After centrifuging the supernatant at a maximum excitation wavelength of 480 nm and a maximum emission wavelength of 596 nm, the fluorescence intensity was evaluated at various Dox concentrations (Figure S1a). The difference in fluorescence intensity achieved a comparatively steady value (Figure S2) when the Dox concentration reached 50 M (Fig. 3a), demonstrating that Dox and RMFs had attained a relative saturation state. Free Dox and the RMFs/Dox were resuspended in RNase-free H_2_O or PBS, and the fluorescence intensity was evaluated at 0 h, 2 h, 4 h, 6 h, 8 h, and 10 h to ascertain the stability of the RMFs loaded with Dox. According to the findings, the RMFs/Dox system’s fluorescence intensity did not change considerably after 10 h (Fig. 3b). These RMFs loaded with Dox remained relatively stable in the PBS and RNase-free H_2_O.

RMFs were micron-sized particles, which had an impact on how well cells absorbed them. Cationic Polyethyleneimine (PEI) reacted with the extremely negative charge on the surfaces of the RMFs via electrostatic adsorption to produce particle size reduction [24]. In order to optimize the PEI concentration and compress the RMFs/Dox, PEI was employed in this investigation (Fig. 3c). The particle size was 278 nm after 2 h of incubation with PEI and RMFs/Dox (Fig. 3d). The change from negative to positive in the surface charges of the particles, which was measured and was found to be between 40 and 50 mV (Fig. 3e), indicated that the RMFs/Dox and PEI had been successfully assembled. Additionally, it is possible that the FND may interact with serum albumin following IV injection due to its highly positive surface charge, which could lead to aggregation and clearance. When PEI was further diluted to 15 mg/mL (PEI-15), the particles showed no additional shrinking. PEI is somewhat hazardous at high concentrations when used as a compression reagent (Figure S3). Evaluation of the functional nucleic acid nanoparticles’ PEI content revealed no extra cell toxicity, confirming the system’s safety and effectiveness.

Fluorescence spectrophotometry was used to determine the Dox encapsulation rate. By measuring the fluorescence intensity at various Dox concentrations, the standard fluorescence-dose curve was discovered. In the range of 0.01 M-200 M, a significant linear connection (R^2^ = 0.9895) between the Dox and the fluorescence intensity was discovered (Figure S4), allowing the Dox concentration to be calculated. The RMFs had a maximum Dox encapsulation rate of 73.8% (Fig. 3f). But using PEI to incubate and centrifugally elute the RMFs/Dox resulted in further Dox loss in the supernatant. An encapsulation FND Dox rate of 18.67% was determined by measuring and calculating the Dox concentration of this fraction. This was higher than the 8.2% observed in earlier studies [30], indicating that FND has a high Dox encapsulation rate. Transmission electron microscopy (TEM) and dynamic light scattering (DLS) were used to assess the FND when the circumstances were improved (Fig. 3 g and 3 h). According to the findings, the FND was present as scattered particles with an estimated 270 nm size and a potential of 40 mV (Fig. 3i).

### Targeting and uptake of FND by SW480 cells

**Fig. 4:**
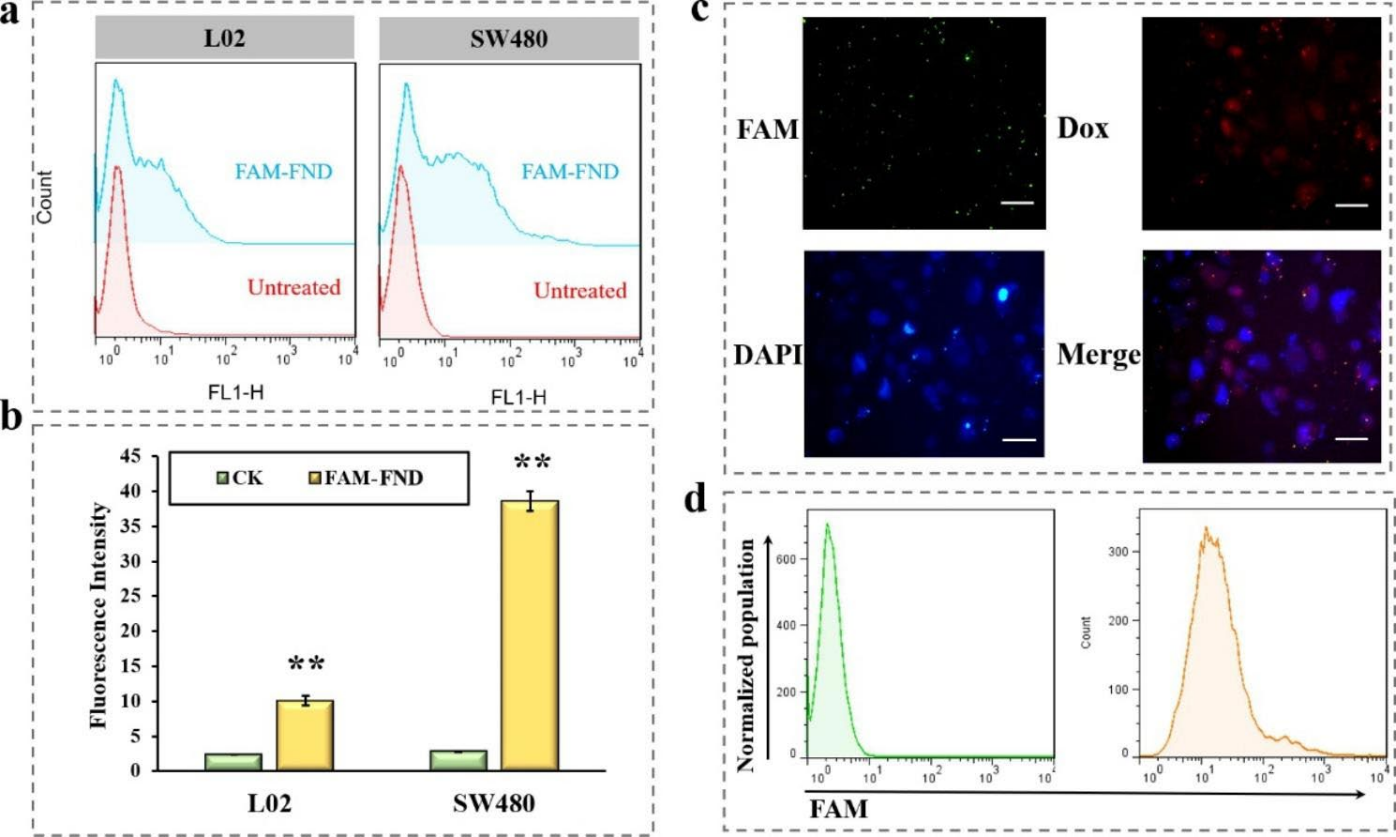
(**a**) Cellular targeting of FND to L02 and SW480 cells. (**b**) Data statistics of targeting detection. (**c**) Uptake of FND by SW480 after 8 h via fluorescence microscope (scale bar: 100 μm). (**d**) Uptake of FND by SW480 before and after 8 h via flow cytometry

Normal human hepatocytes, L02, were chosen as the control group and human colon cancer cells, SW480, as the experimental group to study the cell-targeting effect of FND. The Carboxyfluorescein (FAM)-labeled FND was incubated with L02 and SW480 cells for 8 h, respectively. Following an 8-hour incubation with FND, flow cytometry showed that the initial fluorescence intensity of the cells was 2.3 ± 0.1 for L02 cells and 2.8 ± 0.1 for SW480 cells, respectively, with an average fluorescence intensity of 10.1 ± 0.7 for L02 cells and 38.6 ± 1.4 for SW480 cells (Fig. 4a and b). As AS1411 targets nucleolin, non-targeted L02 cells also displayed modest FND uptake. Although it is expressed on the surfaces of normal cells as well, cancer cell surfaces are where it is mostly expressed. Therefore, L02 cells with poor nucleolin expression also exhibited a certain degree of FND uptake. However, SW480’s fluorescence was more intense, suggesting that the FND had some target recognition abilities and could carry out aptamer-based targeting, proving the FND’s capacity to target SW480 cells.

To explore the FND uptake by SW480, FAM-labelled FND were incubated with SW480 cells for 8 h, and the nuclei were stained with 4’,6-diamidino-2-phenylindole (DAPI). The Dox signal was red surrounding the nucleus, while the FAM signal was green. Additionally, the combined image revealed the presence of yellow fluorescence in the vicinity of the nucleus (Fig. 4c), demonstrating the uptake of FND by the cells. In particular, the blue DAPI signal and the red Dox signal were mixed together. By embedding it in DNA, interfering with transcription, and blocking the production of mRNA, the major Dox mechanism involves the impairment of its usefulness as a template for nucleic acid synthesis. This exerted an anti-tumor effect, indicating that Dox accumulated in the nucleus, and was released by FND upon entry into the cell. Flow cytometry showed that the initial fluorescence intensity of the SW480 cells was 2.8 ± 0.1, reaching 36.0 ± 2.2 after FND incubation for 8 h (Fig. 4d), further demonstrating the FND uptake by SW480. This suggests that FND has the potential to synergistically exert anti-miR-21 and Dox anticancer effects.

### Anti-tumor effect of in vitro FND targeting

**Fig. 5:**
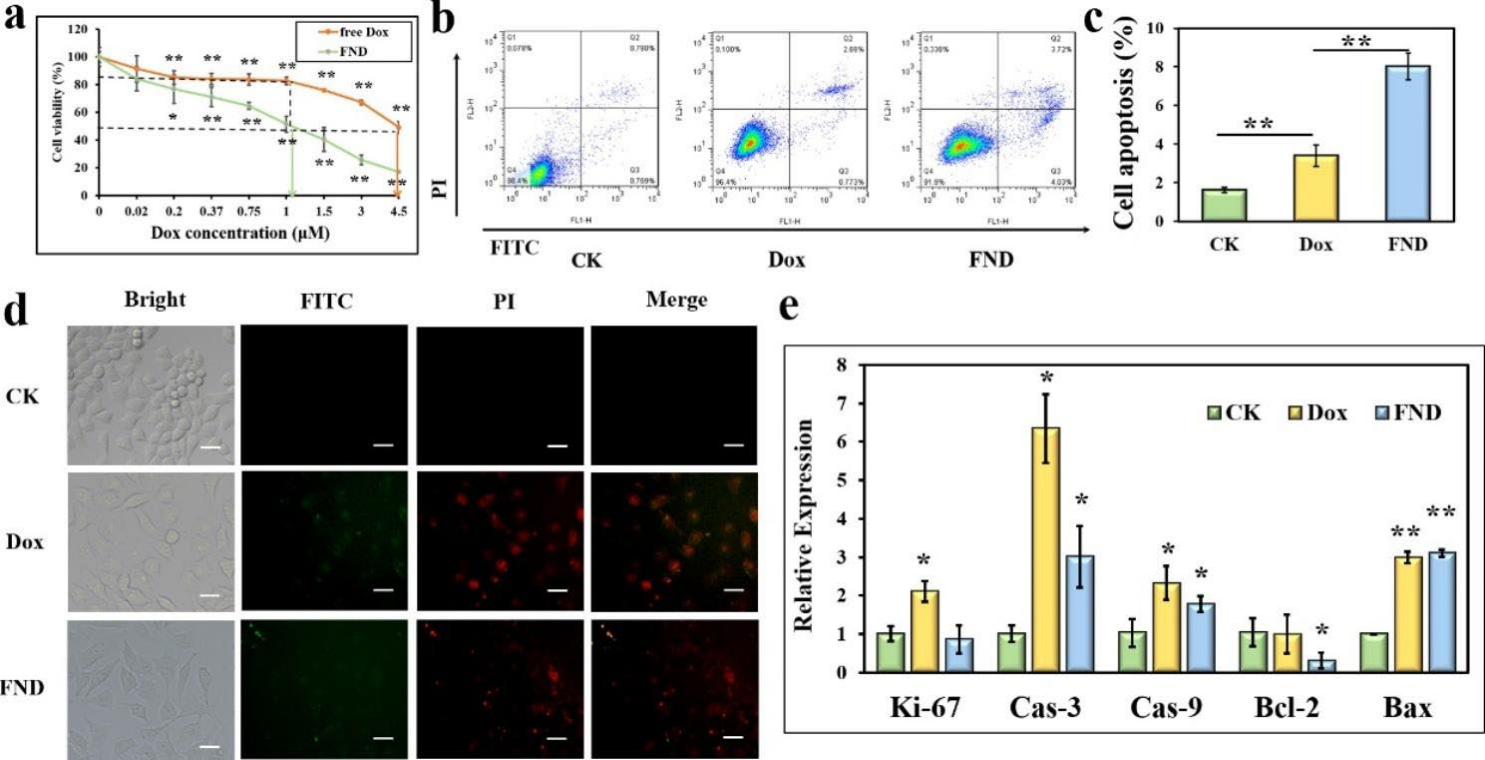
(a) Effect of different concentrations of Dox and FND on the viability of SW480 cells (*p < 0.05, **p < 0.01). (b and c) Flow cytometry detection of cell apoptosis induced by FND (*p < 0.05, **p < 0.01). (d) Fluorescence microscopy detection of cell apoptosis induced by FND (scale bar: 100 μm). (e) Changes in the expression of genes related to cell proliferation and apoptosis induced by FND (*p < 0.05, **p < 0.01)

Free Dox was employed as the control, and SW480 was subjected to different concentrations (0-4.5 M) gradients of free Dox and FND, respectively, for 24 h, to assess the lethality of FND on SW480 cells and to establish the concentration of FND-treated cells in subsequent experiments. The encapsulated Dox concentration served as the foundation for the FND concentration. As the quantities of free Dox and FND rose in a dose-dependent way, cell viability reduced. The IC_50_ of the FND was approximately 1 µM, and the cell viability was 82.67% at a free Dox concentration of 1 µM, while the IC_50_ of the free Dox reached approximately 4.5 µM (Fig. 5a). The findings demonstrated that FND was fatal to SW480 at concentrations lower than free Dox, suggesting promise as a synergistic treatment approach since it lessened the toxic effects of chemotherapeutic medicines. Finally, 1 µM was selected as the cell treatment concentration for both FND and free Dox.

In this study, the apoptosis that Dox and FND cause in SW480 cells was also examined. In contrast to the 1.61% rate in the control group, the apoptosis rate considerably increased to 3.39% and 8.03% in the Dox and FND groups, respectively (Fig. 5b and c). The fact that the FND group’s apoptosis rate was considerably different from that of the Dox group suggests that FND greatly increased apoptosis in the SW480 cells. The results of the fluorescence microscopy showed that the morphology of the SW480 cells altered dramatically after 24 h of Dox and FND treatment, going from the somewhat rounded morphology of the cells before to treatment to an elongated, diamond-shaped morphology. Additionally, both the Dox and FND groups had red PI and green Annexin-V FITC fluorescence (Fig. 5d). This revealed that considerable SW480 apoptosis was induced by FND, which was consistent with the flow cytometry findings. Furthermore, this study examined the expression of some proliferation- and apoptosis-related genes in SW480. The proliferation-related gene *Ki-67* is strongly linked to the expansion of several types of cancer cells and is essential for the development and growth of tumors. By lowering homogenous intercellular adhesion, fostering tumor angiogenesis, and altering the extracellular matrix, it may also encourage tumor spread and invasion [31]. Apoptosis is a process of programmed cell death regulated by a variety of genes, while caspases and Bcl-2 play important regulatory roles in tumor cell apoptosis. *Caspase-3* and *caspase-9* cause apoptosis and play an important role in tumorigenesis and development [32, 33]. Bcl-2 includes the most representative apoptosis suppressor gene, *Bcl-2*, and the most representative apoptosis promoting gene, *Bax* [34, 35]. *Ki-67* expression in SW480 cells fell somewhat but not dramatically after 24 h of FND treatment, but *caspase-3*, *caspase-9*, and *Bax* expression levels were markedly up-regulated, and *Bcl-2* expression was markedly down-regulated (Fig. 5e). These findings added to the evidence that FND caused apoptosis.

**Fig. 6:**
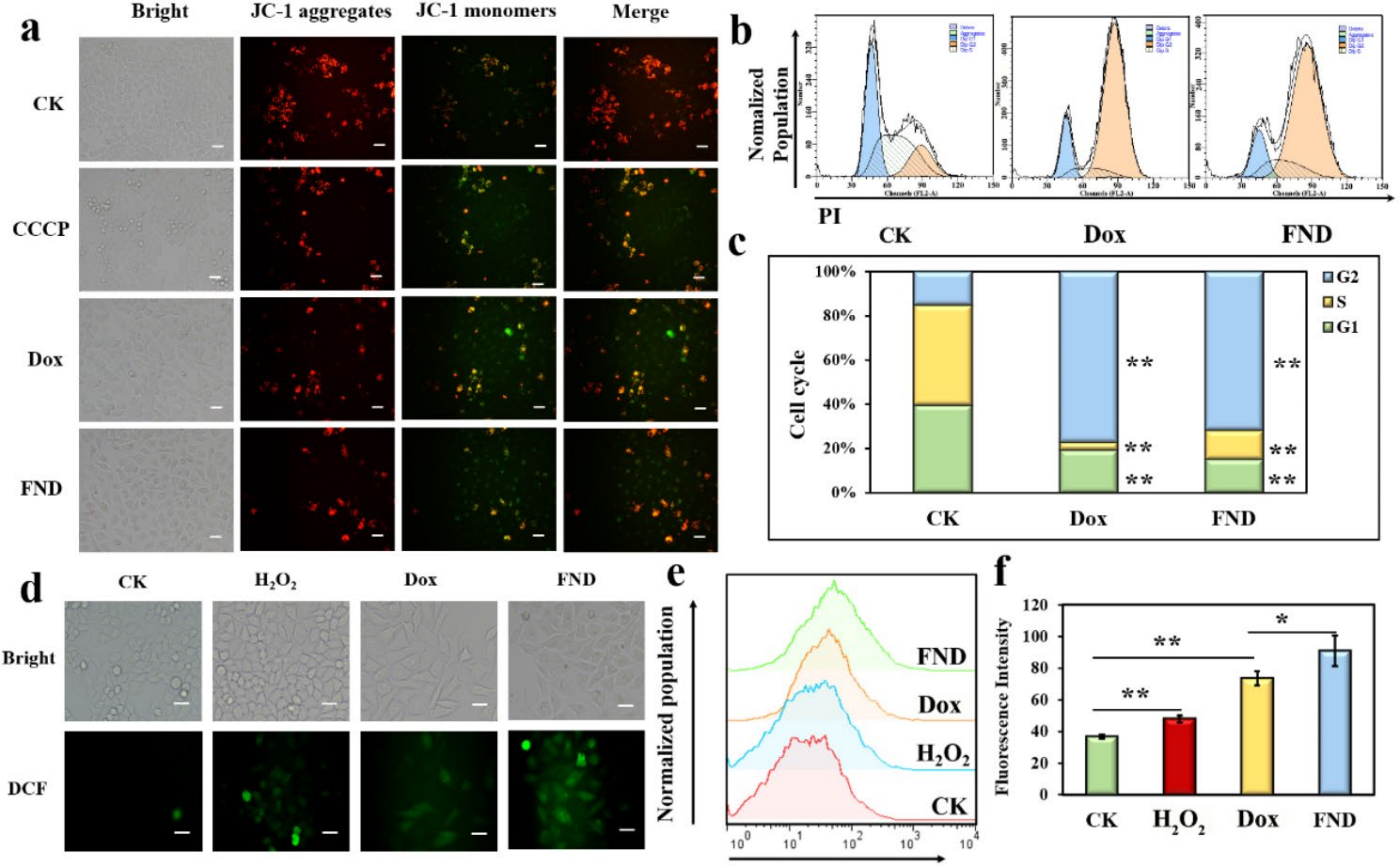
(**a**) Fluorescence microscopy detection of FND-induced decrease in cell membrane potential (scale bar: 100 μm). (**b** and **c**) Flow cytometry detection of cell G2/M cycle arrest induced by FND (*p < 0.05, **p < 0.01). (**d**) Fluorescence microscopy detection of FND-induced increase in cellular ROS (scale bar: 100 μm). (**e** and **f**) Flow cytometry detection of FND-induced increase in cellular ROS (*p < 0.05, **p < 0.01)

Using a JC-1 probe, the ΔΨm of the cells treated with FND for 24 h was assessed in order to describe the influence of FND on mitochondrial membrane potential (MMP) modulation. In the mitochondrial matrix, this probe aggregated to create JC-1 polymers (red) at high MMP and JC-1 monomers (green) at low MMP. The results showed significant green fluorescence in the apoptosis-inducer Carbonyl cyanide 3-chlorophenylhydrazone (CCCP), Dox, and FND groups compared to the control, indicating the production of JC-1 monomers (Fig. 6a). This proved that FND caused the MMP in the SW480 cells to drop, proving that early apoptosis had taken place.

This investigation also looked at how FND affected the cell cycle. PI dye was used to mark the cells, and flow cytometry was used to calculate the relative concentration of intracellular DNA. The percentages of cells in the G2 phase increased in the Dox and FND groups relative to the control group from 15.12 to 77.16% and 71.74%, respectively, while the percentages of cells in the G1 and S phases decreased from 39.42 to 19.29% and 14.94%, respectively (Fig. 6b and c). These results demonstrated that SW480 cells were primarily in the G2/M phase following Dox and FND treatments. These findings demonstrated that FND caused G2/M phase arrest in the SW480 cells, indicating aberrant cell growth. In other words, FND could inhibit the cell proliferation of SW480 by inducing cell cycle arrest.

The impact of FND therapy on the ROS levels in SW480 cells was also investigated in this study. The H_2_O_2_, Dox, and FND groups showed a significantly higher mean fluorescence intensity than the control group (Fig. 6d). The fluorescence intensity of the FND group was 91.0 ± 9.7, which was substantially higher than that of the Dox group at 73.5 ± 4.5 (Fig. 6e and f). According to consistent findings from flow cytometry and fluorescence microscopy, FND caused oxidative damage in the SW480 cells by causing an intracellular ROS burst.

Finally, *miR-21* expression was discovered. After 24 h of Dox treatment, *miR-21* expression in SW480 considerably increased in comparison to the control group, however after 24 h of FND treatment, there was no discernible difference (Figure S5). Additionally, *miR-21* expression was significantly lower in the FND group compared to the Dox-treated group under the same concentration conditions (same 1 µM Dox), indicating that anti-miR-21 in FND played a role and that Dox and anti-miR-21, the functional components of FND, could have a synergistic effect on SW480.

### Transcriptomics reveal the potential mechanism behind anti-tumor effect of FND

**Fig. 7:**
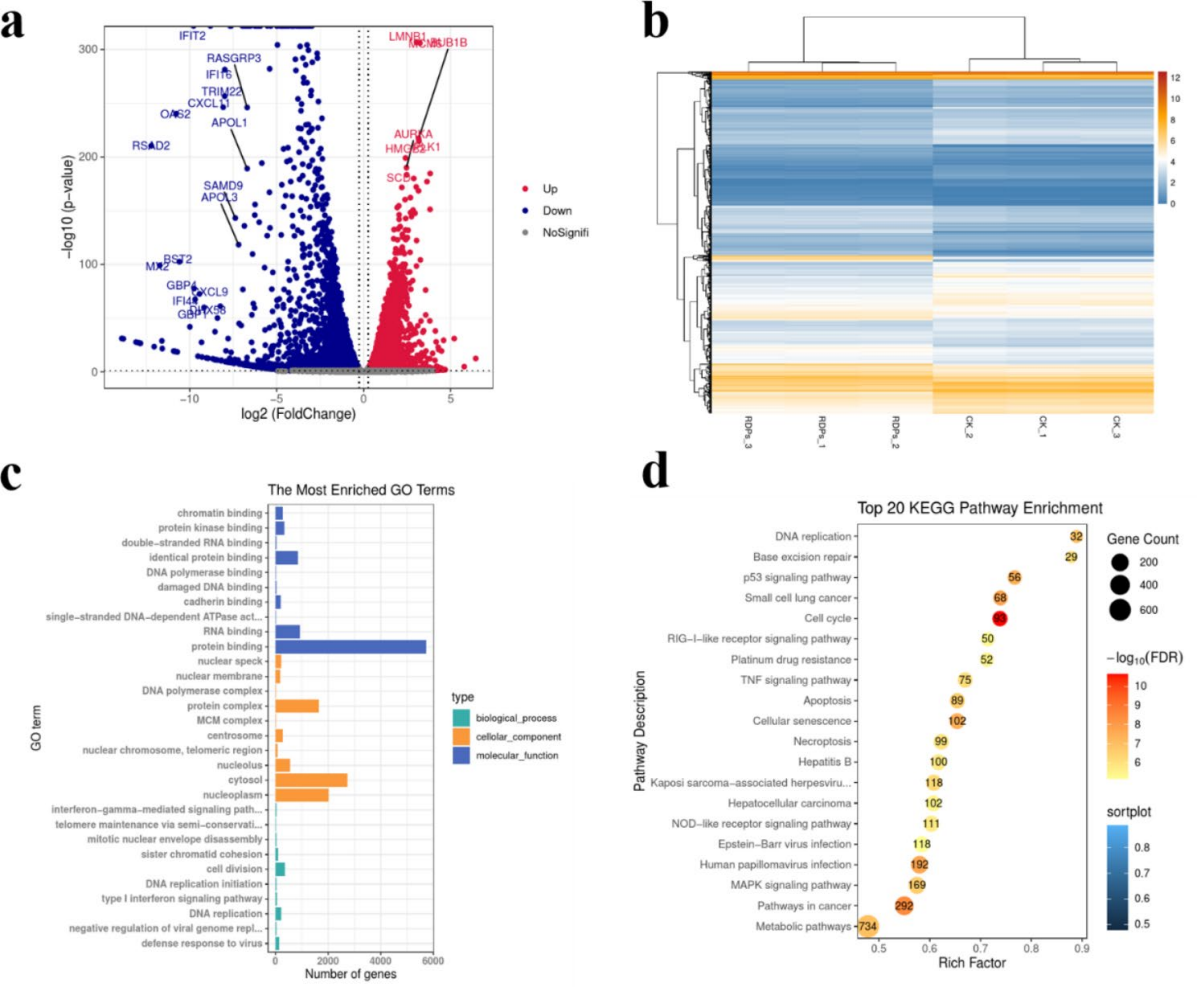
(**a**) Cluster analysis of DEGs in volcanoes. (**b**) Heat map of clustering analysis of DEGs. (**c**) GO enrichment pathway analysis of DEGs. (**d**) KEGG enrichment pathway analysis of DEGs.

Transcriptomics analysis was performed on the SW480 cells in the control and FND groups in order to investigate the probable mechanism underlying FND-induced cytotoxicity. First, a total of 9356 differentially expressed genes (DEGs, *p* ≤ 0.05 and log_2_|fold change| > 1) were identified in the FND and CK groups, of which 4562 were up-regulated and 3991 were down-regulated. Their distribution is shown in Fig. 7a and b. As a result, giving the SW480 cells a treatment of 1 µM FND for 24 h drastically altered their gene expression profile. Table 1 displays the top 20 DEGs.

**Table 1:** The top 20 DEGs

Gene-ID	Log_2_(fold-change)	*p*-value	Change
BCAR1	-0.263480991	0.010122	Up
KLHDC10	-0.263528783	0.004392	Up
NAIF1	-0.263602452	0.044304	Up
ASB8	-0.263644472	0.029631	Up
FBXO28	-0.263969334	0.00876	Up
MED6	-0.26406292	0.006429	Up
GOLPH3L	-0.264197449	0.008392	Up
PFKL	-0.264223736	0.00406	Up
SGPL1	-0.264599955	0.002325	Up
ASH1L	-0.264773015	0.014215	Up
SLC25A48	6.437223	3.03e-13	Down
C3orf80	5.769758	1.33e-05	Down
C2CD4C	5.200656	1.08e-31	Down
SCN1B	4.677231	0.007549	Down
RP11-715J22.2	4.670051	0.008758	Down
PALM3	4.589662	4.15e-30	Down
FXYD7	4.568897	0.016772	Down
RP11-728G15.1	4.529956	0.002361	Down
CACNG4	4.485656	0.012373	Down
NRTN	4.470327	4.00e-20	Down

Gene Ontology (GO) and the Kyoto Encyclopedia of Genes and Genomes (KEGG) databases were used to examine the DEGs. The three categories considered in the GO enrichment analysis were biological processes (BP), molecular functions (MF), and cellular components (CC). In contrast to CC, which focused on cytosol, nucleoplasm, and protein complex components, the MF-related enrichment analysis primarily focused on protein binding and RNA binding (Fig. 7c). Cell division and DNA replication were the main topics of the BP-related enrichment study (Fig. 7c). The KEGG database was used to find the top 20 signaling pathways. Cell cycle, cancer, cellular senescence, and p53 signaling pathways were the main signaling pathways involved (Fig. 7d).

**Fig. 8:**
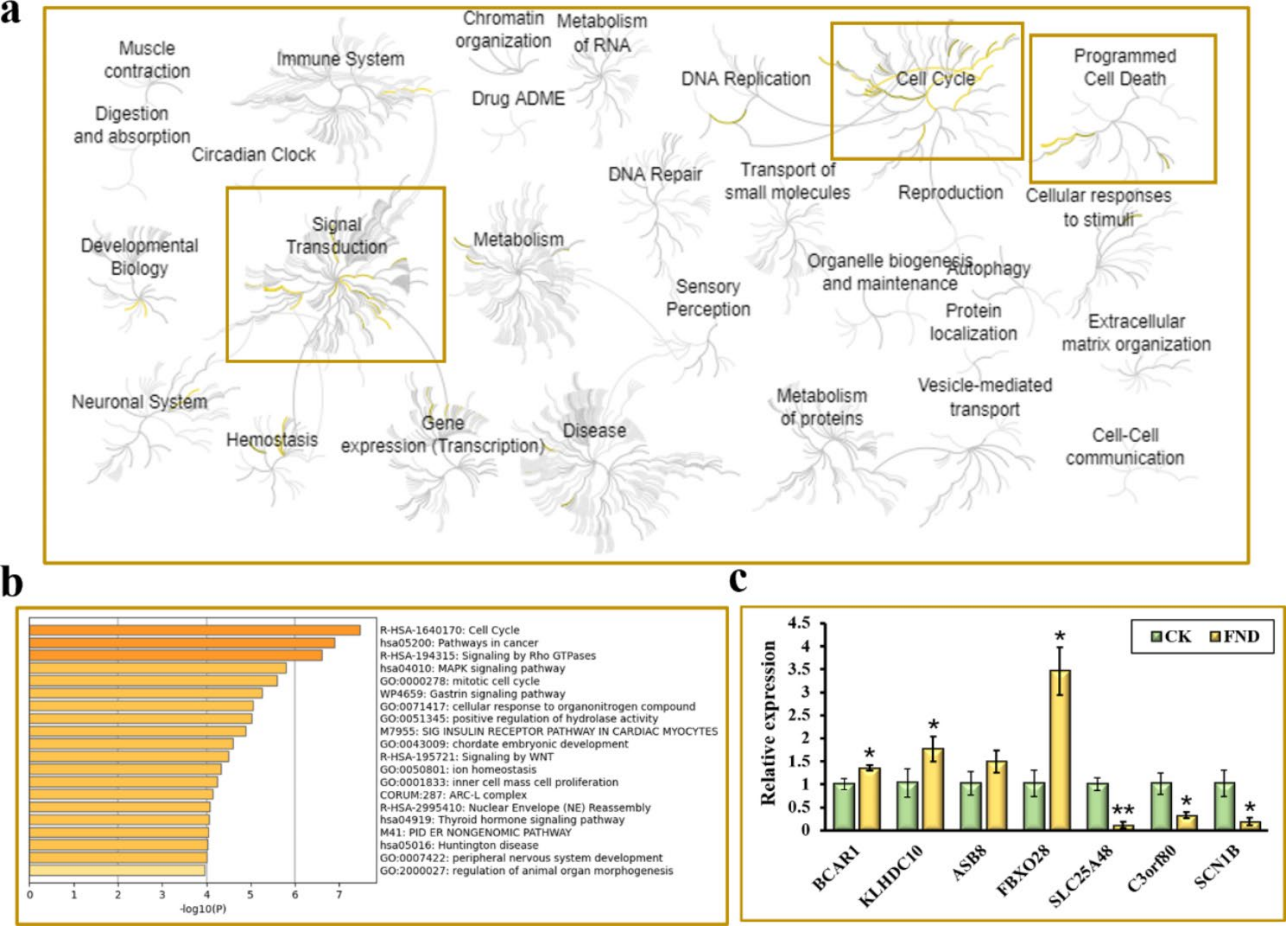
(**a**) Reactome enrichment pathway analysis of DEGs. (**b**) Metascape enrichment pathway analysis of DEGs. (**c**) The mRNA expression by Real-time PCR after FND treatment (*p < 0.05, **p < 0.01)

In addition, the Reactome and Metascape databases (https://reactome.org and https://metascape.org) were used to analyze the signaling pathways involving DEGs. The top 200 DEGs and the related pathways were analyzed, 20 of which were mainly enriched via the Reactome database. The top 5 signaling pathways included G-protein beta: gamma signaling, gene and protein expression via JAK-STAT signaling after interleukin-12 stimulation, mitotic anaphase, mitotic metaphase, and interleukin-12 signaling (Fig. 8a). Of these 20 pathways, those associated with the cell cycle included mitotic anaphase and the phosphorylation of APC/C. The signaling pathways associated with apoptosis included the SMAC (DIABLO)-mediated dissociation of IAP: caspase complexes, and caspase activation via apoptosome-mediated cleavage. The signaling pathways related to signal transduction included G-protein beta: gamma signaling, β-catenin formation: TCF transactivating complex, G beta: gamma signaling via CDC42, and the Wnt signaling pathway (Fig. 8a).

The top 20 signaling pathways were identical to those enriched using the Reactome database, according to the enrichment assessment of the top 200 DEGs via the Metascape database. Genes and enriched pathways were revealed to be correlated and to interact with one another (Figure S6). The cell cycle, the mitotic cell cycle, and nuclear envelope reassembly were all linked to it (Fig. 8b). Hydrolase activity modulation was one of those connected to cell death (Fig. 8b), whereas the Rho GTPase, MAPK, and Wnt signaling pathways were those connected to signal transduction.

Furthermore, to verify the accuracy and reliability of the transcriptomics results, seven DEGs were selected for real-time polymerase chain reaction (PCR). The *BCAR1*, *KLHDC10* and *FBXO28* was significantly up-regulated (Fig. 8c), while that of *SLC25A48*, *C3orf80*, and *SCN1B* was considerably down-regulated (Fig. 8c), which was consistent with the transcriptomics results, confirming their accuracy and reliability.

## Conclusion

This study develops an NSD for the targeted delivery of AS1411, antimiR-21, and Dox to treat colon cancer. This nanosystem is assembled via RCT to obtain the AS1411 aptamer and antimiR-21 sponge balls. Dox is skillfully embedded between the -G-C-base pairs on the sponge ball to achieve nano-synergistic therapeutic system assembly (AS1411@ antimiR-21@ Dox). Additionally, a large number of AS1411 aptamers directed the NSD’s distribution to cancer cells. An in vitro cell model is used to assess the lethality of FND by looking for changes in cell viability, cell apoptosis, cell cycle, ROS concentration, and MMP. Transcriptomics also reveals FND’s anti-tumor effect via mitotic metaphase and anaphase, as well as SMAC-mediated dissociation of IAP: caspase complexes. Therefore, this system displays the potential for the synergistic treatment of colon cancer by inducing cell cycle arrest and cell apoptosis.

## Materials and methods

### Materials

The T4 DNA ligase, 10×T4 DNA ligase buffer, T7 RNA polymerase, and 10×T7 RNA polymerase buffer were purchased from Novoprotein Scientific Inc. (Suzhou, China). The phi29 DNA polymerase and 10×phi29 DNA polymerase buffer were obtained from New England Biolab (UK). The RNase inhibitor, ethidium bromide (EB), DNA Ladder, RNase-free H_2_O, *TransScirpt®* One-Step gDNA Removal and cDNA Synthesis SuperMix and RealMsterMix(SYBR Mix)were purchased from Tiangen Biotech Co. Ltd. (Beijing, China). The DTT and dNTP Mixture were obtained from Takara (Beijing, China), while the rNTP Mixture and SYBR Gold were purchased from Thermo Fisher Scientific (MA, US). The Tris-base and ethylene diamine tetraacetic acid (EDTA) were obtained from Biotopped Life Sciences (Beijing, China). The sodium chloride (NaCl), sodium monohydrogen phosphate (Na_2_HPO_4_), monopotassium phosphate (KH_2_PO_4_), and dimethylsulfoxide (DMSO) were supplied by Sigma-Aldrich (St. Louis, MO). The SW480 cells, human colon cancer cells, and L02 cells, normal human hepatocytes, were acquired from the National Experimental Cell Resource (BMCR) sharing platform (Beijing, China). The Dox and Dulbecco’s Modified Eagle Medium (DMEM) were obtained from Solarbio Life Sciences (Beijing, China), while the fetal bovine serum (FBS) and Trypsin-EDTA (0.25%) were purchased from Gibco (USA). The Cell Counting Kit-8 (CCK-8), Cell Cycle and Apoptosis Analysis Kit, Annexin V-FITC Cell Apoptosis Kit, MMP assay kit with JC-1, and ROS Assay Kit were obtained from Beyotime (Beijing, China). All DNA sequences were synthesized by Sangon Biotech (Shanghai, China).

### Construction of circular template DNA

The hsa-miR-21 sequence was queried against the miRNA database, miRBase (http://www.mirbase.org/index.shtml). The AS1411 aptamer and T7 promoter sequences were obtained from available references [19, 21, 36] (Table S2). This study designed the DNA template, promoter, and Apt-DNA sequences. The cyclization of the single-stranded DNA template was then carried out. The DNA template, promoter, 10×T4 DNA ligase buffer, and RNase-free H_2_O were added to 200 µL EP tubes and mixed, placed in a PCR instrument at 95 °C for 10 min, and cooled to 25 °C at a 1 °C/min gradient. Next, 4.8 µL of T4 DNA ligase (40 U/µL) was added to the system, mixed thoroughly, and placed in a PCR machine overnight at 16 °C to obtain the ligated product. The product was then mixed with phi29 DNA polymerase, 10×phi29 DNA polymerase buffer, and RNase-free H_2_O and placed in a PCR machine for 2 h at 30 °C. The product was collected and stored at 4 °C. The circle template products were verified via 2% agarose gel electrophoresis.

### Optimization of the RCT conditions

The circular template concentrations, T7 RNA polymerase, DTT, and PCR reaction time were optimized for maximum RCT results. Here, the rNTP, RNAase inhibitor, RNA polymerase buffer, and T7 RNA polymerase were mixed in an RNase-free 200 µL EP tube. Water was added to the final volume and vortexed gently to mix well. The mixture was placed in a PCR instrument at 37 °C, after which 6.4 µL DNA-Apt was added at 65 °C for 5 min and lowered to room temperature at a 2 °C/min gradient, followed by storage in a refrigerator at 4 °C for 2 h. The product was washed with RNase-free H_2_O via centrifugation (4 °C, 12,000 g, 30 min) and resuspended to its original volume with RNase-free H_2_O. The circle template concentrations were set to 0.25 µM, 0.5 µM, 1 µM, and 2.5 µM, while those of the T7 RNA polymerase was 2.5 U/µL and 5 U/µL, respectively. The final DTT concentrations in the T7 RNA polymerase buffer were 1 mM, 2.5 mM, and 5 mM, respectively, while the PCR reaction times were 8 h, 16 h, 24 h, and 48 h, respectively. Finally, the circle template products were analyzed via 2% agarose gel electrophoresis to determine the RCT conditions.

### Verification of the RCT products

The SYBR Gold dye was diluted with 1 x TBE and incubated with the product for 10 min, protected from light, and washed via centrifugation with RNase-free H_2_O (4 °C, 12,000 g, 30 min). The process was finished by dropping the sample onto a slide and applying nail polish on the coverslip. After that, the item was examined using a fluorescence microscope and blue light excitation.

### DLS analysis

The FND was diluted with RNase-free H_2_O in appropriate amounts and sonicated for 2 min for thorough dispersion. Particle size and potential cuvettes received the samples, accordingly. Using a Nano ZS90, the samples’ potential and particle size distribution were assessed.

### SEM analysis

The product was washed with RNase-free H_2_O, centrifuged (4 °C, 12,000 g, 30 min), resuspended in RNase-free H_2_O, and sonicated for 2 min to disperse well. A drop of 3 µL was added to the center of the smooth surface of a clean 5 × 5 mm single-polished silicon wafer and placed in a metal bath at 37 °C to dry. Prior to detection, the sample was gold-sprayed onto the wafer. The product morphology and size were evaluated using 2 kV electron microscopy scanning.

### EDS analysis

An SEM fitted with an EDS detector was used to carry out the EDS characterization. The samples were made by adding each component dropwise to the middle of a clean, single-throw silicon wafer, then drying them at 37 °C in a metal bath. A non-standard quantitative method was employed to determine the relative atomic ratio normalized to each particle’s Mg content for the EDS examination.

### Preparation of the FND

The product was mixed with different Dox concentrations (1 µM, 5 µM, 10 µM, 20 µM, 50 µM, and 100 µM). At the same time, the same volume of RNase-free water was mixed with Dox concentrations (1 µM, 5 µM, 10 µM, 20 µM, 50 µM, and 100 µM). Then, these samples were incubated on a shaker in a cold room at 4 °C for 2 h. The samples were then centrifuged at high speed (4 °C, 12,000 g, 30 min), and the supernatant was used to determine fluorescence intensity using a microplate reader (ELx808, USA). The difference between the two groups of fluorescence intensity was taken to make a line chart. When the difference value is in a relatively stable stage, it indicated that the loading of products on Dox has reached a relatively saturated state.

### Encapsulation efficiency and loading capacity of the FND

To characterize the stability of Dox loading in RNase-free H_2_O and PBS, free Dox and products loaded with Dox were resuspended in RNase-free H_2_O or PBS, respectively, while the fluorescence intensity was monitored at 0 h, 2 h, 4 h, 6 h, 8 h, and 10 h, respectively, using a microplate reader (ELx808, USA).

A fluorescence spectrophotometer was employed to measure the fluorescence intensity of the different Dox concentrations (0.01 µM, 0.1 µM, 1 µM, 5 µM, 10 µM, 100 µM, and 200 µM) and create a standard curve. The Dox encapsulation rate was calculated using the following formula: Loading capacity of Dox= (total Dox amount- Dox amount in the supernatant)/total Dox amount×100.

### Characterization of the FND

The FND was characterized using DLS and TEM. The DLS procedure was the same as mentioned above. The FND was washed with RNase-free H_2_O, centrifuged (4 °C, 12,000 g, 30 min), resuspended in RNase-free H_2_O, and sonicated for 2 min to disperse well. A small amount of the sample solution was dipped in a copper mesh, blown dry with nitrogen, and observed via TEM.

### Cell culture

The SW480 and L02 cell medium consisted of DMEM basal medium containing 10% (v/v) FBS, 100 U/mL penicillin, and 100 U/m streptomycin. The cells were cultured at 37 °C in 5% CO2 and 95% saturated atmospheric humidity conditions. Cell passaging was performed via 0.25% trypsin digestion. For cell seeding, the cells were inoculated into 96-well, 6-well, or 24-well plates for 24 h, according to the experimental requirements. When the cells reached 70% confluence, the cells were treated with FND or Dox, followed by subsequent experiments.

### FND targeting

The FND was prepared using a FAM-labeled aptamer (FAM-Apt) to obtain fluorescently labeled FAM-FND. The SW480 and L02 cells were seeded into 6-well plates, while control and FAM-FND groups were established, with three replicate wells in each group. The cells were treated with FAM-FND for 8 h when they reached 70% confluence. Finally, the cells were resuspended in PBS, filtered through a 40 μm cell strainer into a flow tube, and examined via flow cytometry.

### FND uptake

The SW480 and L02 cells were seeded into 24-well plates and treated with FAM-FND for 8 h when they reached 70% confluence. The culture medium was discarded, after which the cells were washed once with PBS and incubated with DAPI at 37 °C for 5 min. The working solution was discarded, and the cells were washed, after which they were examined via fluorescence microscopy and flow cytometry for detection.

### Cell viability

The SW480 cells were seeded into a 96-well plate for 24 h. When they reached 70% confluence, different FND or Dox concentration gradients (Dox concentration: 0 µM, 0.02 µM, 0.2 µM, 0.37 µM, 0.75 µM, 1 µM, 1.5 µM, 3 µM, and 4.5 µM) of 100 µL culture solution was used to treat the cells for 24 h. 10 µL of CCK8 was added into each well, mixed, and incubated at 37 °C for 1 h. The absorbance was measured at 450 nm using a microplate reader (ELx808, USA). The cell viability was calculated using the following formula: Cell viability (%) = (OD_sam_-OD_blank_)/(OD_ck_-OD_blank_) ×100.

### Cell cycle

The SW480 cells were seeded into 6-well plates. CK, Dox, and FND groups were established. Cells were treated with FND or Dox for 24 h once they had achieved 70% confluence, then collected after being washed and digested. The cells were then rinsed with pre-cooled PBS and centrifuged once more. The precipitated cell material was then resuspended in 1 mL of pre-cooled 70% ethanol and fixed for an overnight fixation period at 4 °C. The following day, fixed cells were collected by washing in PBS that had already been chilled. To collect the cells, the washing procedure was repeated. The cells were then incubated for 30 min at 37 °C in the dark and subjected to flow cytometry analysis.

### Cell apoptosis

The SW480 cells were seeded into 6-well plates. CK, Dox, and FND groups were established, with three replicate wells per group. When the cells reached 70% confluence, they were treated with FND or Dox for 24 h and washed with PBS, followed by cell digestion and centrifugation to collect the cell precipitate. The cell precipitates were then resuspended using 195 µL Annexin V-FITC conjugate from the Annexin V-FITC assay kit, followed by the addition of 5 µL Annexin V-FITC and 10 µL PI, and mixed well. The cells were incubated for 25 min at room temperature, protected from light, and detected via flow cytometry or observed via fluorescence microscopy.

### ROS content

The SW480 cells were seeded into 6-well plates. CK, H_2_O_2_ positive, Dox, and FND groups were established, with three replicates in each group. After 21 h of treatment, the medium in the positive wells was replaced with fresh medium containing 500 µM H_2_O_2_, and the cells were treated for another 3 h. After collection, the cells were suspended in a fluorescent DCFH-DA probe (10 µM) diluted in serum-free culture, incubated for 20 min at 37 °C, protected from light, washed three times with serum-free culture medium, and prepared for flow cytometry or fluorescence microscopy.

### MMP

The SW480 cells were seeded into 24-well plates. CK, CCCP positive control, Dox, and FND groups were established. After 23 h 40 min of treatment, the medium in the positive control wells was replaced with fresh medium containing CCCP (10 µM), and the cells were treated for 20 min. After treatment, the medium was removed, and the cells were washed once with PBS, followed by the addition of 250 µL of cell culture medium and 250 µL of JC-1 staining solution and incubation at 37 °C for 20 min. After staining, the supernatant was removed, and the cells were washed twice with 1× JC-1 staining buffer, after which 500 µL of cell culture solution was added. The cells were directly observed using a fluorescent microscope.

### Quantitative real-time PCR (qRT-PCR)

After discarding the media from the 6-well plate, 1 mL of Trizol reagent was added, and samples were collected in RNase-free 1.5 mL centrifuge tubes after 10 min of lysis on ice. Each sample tube received 200 µL of trichloromethane after the samples were lysed on ice for an additional 10 min. The samples were well mixed, vortexed, and placed on ice for 15 min before being centrifuged at 4 °C and 12,000 g for that same period of time. Each tube was then filled with 500 µL of isopropanol, mixed, and kept on ice for 20 min before being centrifuged at 4 °C at 12,000 g for 20 min and being allowed to air dry at ambient temperature. The RNA was dissolved with RNase-free water, and its concentration was determined using Nanodrop. Finally, the RNA was stored at -80 °C.

U6 and Oligo(dT) were selected as the reference genes for miRNA and mRNA quantification (Table S3). Here, the 20 µL reverse transcription system for miRNA consisted of 500 ng RNA, 0.5 µL RT-primer (10 µM), 10 µL 2×TS Reaction Mix, 1 µL TransScript RT/RI Enzyme mix, 1 µL gDNA Remover, and RNase-free H_2_O to 20 µL. The 20 µL reverse transcription system for mRNA consisted of 2 µg RNA, 0.5 µL oligo (dT), 10 µL 2×TS Reaction Mix, 1 µL TransScript RT/RI Enzyme mix, 1 µL gDNA Remover, and RNase-free H_2_O to 20 µL. The reverse transcription program consisted of 42 °C for 15 min and 85 °C for 5 s.

The 20 µL PCR system for miRNA and mRNA included 2x SuperReal PreMix Plus (SYBR GREEN), a forward primer (10 µM), a reverse primer (10 µM), cDNA, and RNase-free H_2_O. The PCR program comprised the following 95 °C for 5 min, 95 °C for 10 s, 58 °C for 40 s, and 72 °C for 10 s, with 40 cycles. The melting curve was detected, and β-actin was used for mRNA normalization. The RT-PCR primers of all the mRNAs are listed in Table S4. The relative gene expression was calculated as 2^−ΔΔCt^. Each sample was detected in triplicate.

### mRNA library construction and sequencing

The mRNA was isolated from the total RNA using RNA binding buffer, wash buffer, Tris buffer, and Mix I, after which cDNA synthesis was performed according to the protocol. The concentration was measured using Qubit, while the subsequent library was constructed using a KAPA Hyper Prep Kit for Illumina. End repair was performed first, followed by junction ligation and finally elution with 30 µL nuclease-free H_2_O, after which the concentration was measured using Qubit. The constructed libraries were sequenced on the Illumina HiSeq X Ten platform.

### Bioinformatics analysis

After sequencing, the raw data was filtered and processed. Following quality evaluation and qualification, each one was compared with a particular reference gene before being matched with the genome to provide mapped reads. Following that, base region distribution and homogeneity analyses were used to gauge the quality of the library sequences. After analyzing the differentially significant genes, the DEGs were screened using fold changes ≥ 1.2 or ≤ 1.2 and q < 0.05. In order to annotate the DEGs and correct p < 0.05 as the threshold of significance, the genomes were examined using the GO, KEGG, Reactome, and Metascape databases.

### Statistical analysis

The data were shown as mean ± standard deviation (SD). In this study, *P < 0.05 and **P < 0.01 were considered statistically significant by using Student’s t-test (two-tailed) analysis.

## Electronic supplementary material

Below is the link to the electronic supplementary material.

Supplementary Material 1

## Data Availability

The raw data used and/or analyzed in this research are available from the corresponding author upon reasonable request.
