# Supplementary material for: Aptamer functionalized nucleic acid nano drug for targeted synergistic therapy for colon cancer
Source: J Nanobiotechnology. 2023 Jun 7;21:182. doi: 10.1186/s12951-023-01941-z (PMC10245494; doi:10.1186/s12951-023-01941-z)
Supplement: Supplementary file 1 — Supplementary Material 1 [file 12951_2023_1941_MOESM1_ESM.docx]

**Aptamer functionalized nucleic acid nano drug for targeted synergistic therapy for colon cancer**

Liye Zhu^a,c#^, Jieyu Yuhan^a,b#^, Hao Yu^a,b^, Boyang Zhang^a^, Longjiao Zhu^a^, Xiaoyun He^b^,

Kunlun Huang^b^, Wentao Xu^a,b*^

*Corresponding author: Wentao Xu, xuwentao@cau.edu.cn

a Key Laboratory of Precision Nutrition and Food Quality, Department of Nutrition and Health, China Agricultural University, Beijing 100083, China

b College of Food Science and Nutritional Engineering, China Agricultural University, Beijing, 100083, China

c College of Veterinary Medicine, China Agricultural University, Beijing, 100094, China

#Contributed equally

Address: China Agricultural University, No. 17 Qinghua Donglu, Beijing, China, 100083


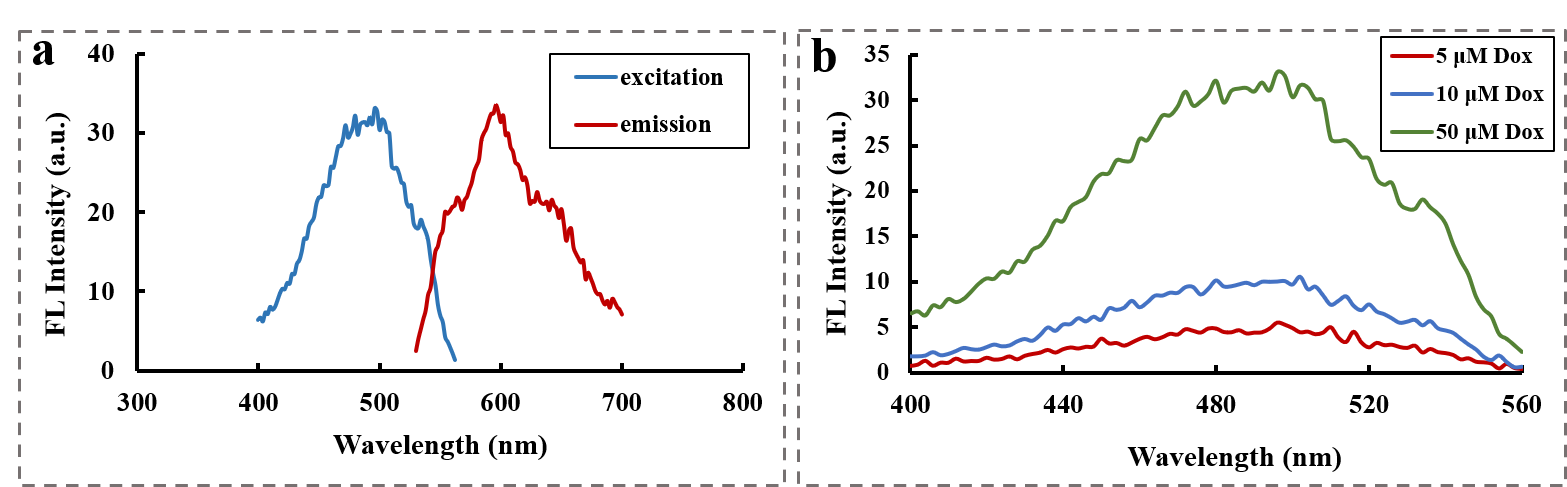


**Figure S1** (a) Maximum excitation and emission wavelength of Dox determined. (b) Emission wavelength scanning of Dox at different concentrations.


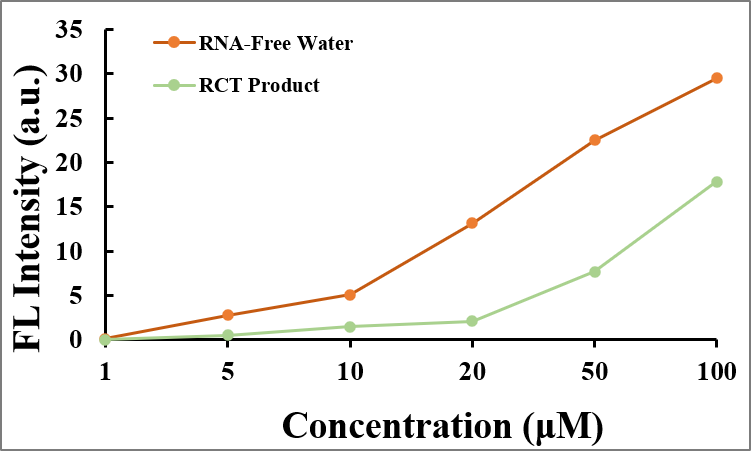


**Figure S2** The fluorescence intensity of Dox in the supernatant


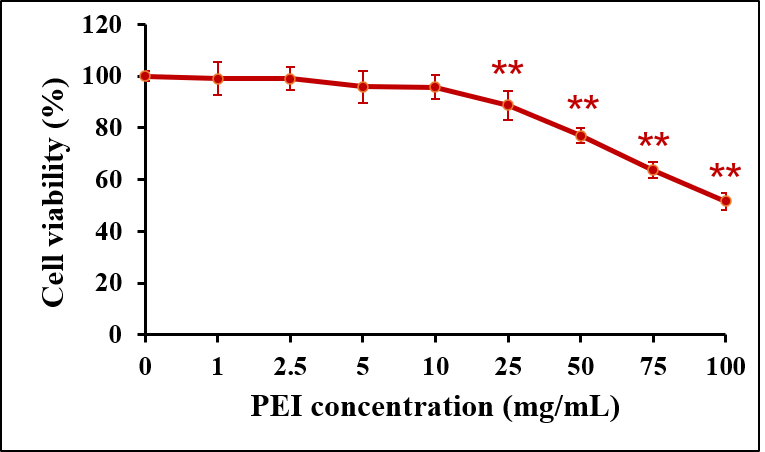


**Figure S3** Effect of different concentrations of PEI on the viability of SW480 cells (**p<0.01)


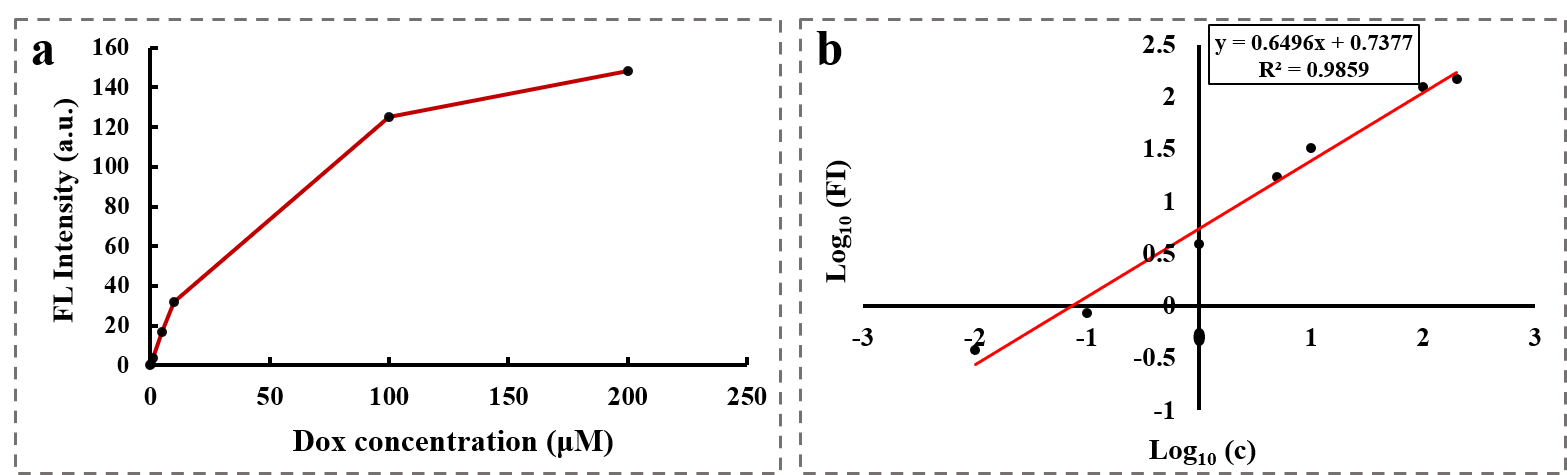


**Figure S4** (a) Fluorescence intensity of Dox at different concentrations. (b) Dox standard curve based on the fluorescence intensity of Dox at different concentrations by logarithmic conversion.


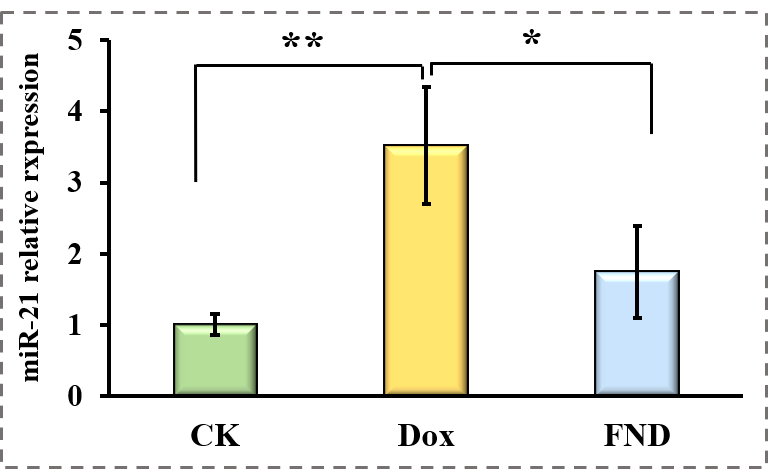


**Figure S5** The expression of *miR-21* after FND treatment (**P*<0.05, ***P*<0.01)


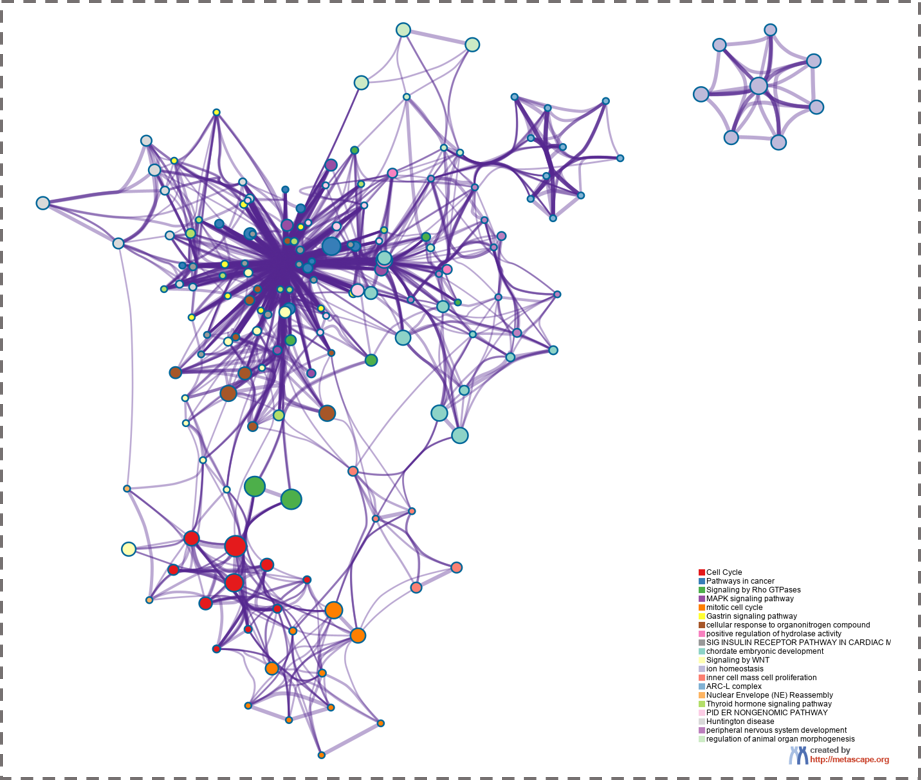


**Figure S6** Visual clustering of inter-gene and inter-pathway interactions by Reactome enrichment pathway analysis of DEGs.

**Table S1** The effect of time on RCT

| **Time (h)** | **Size (nm)** | **PdI** | **Potential (mV)** |
| --- | --- | --- | --- |
| 8 | 250±8 | 0.37±0.01 | -18.4±0.2 |
| 16 | 921±24 | 0.55±0.06 | -18.3±1.2 |
| 24 | 1104±25 | 0.55±0.04 | -19.9±1.5 |
| 32 | 1106±177 | 0.62±0.08 | -19.1±0.9 |

**Table S2** The information of sequence

| Name | Sequence (5’-3’) |
| --- | --- |
| DNA template | 5’-phosphoraylated-ATAGTGAGTCGTATTAACGTACCAATAGCTTATCAGACTGATGTTGACTTGCAACATCAGTCTTTATCTTAGAGGCATATCCCT-3’ |
| Promoter | TAATACGACTCACTATAGGGAT |
| Apt-DNA | TTTATCTTAGAGGCATATCCCTAAAAAAAAAGGTGGTGGTGGTTGTGGTGGTGGTGG |

**Table S3** Reverse transcription primers of miRNAs

| Name | Sequence (5’-3’) |
| --- | --- |
| *hsa-miR-21* | CTCAACTGGTGTCGTGGAGTCGGCAATTCAGTTGAGTCAACATC |
| *hsa-U6* | AACGCTTCACGAATTTGCGT |

**Table S4** Real-time PCR primers of miRNAs and mRNAs

| Name | Forward primer (5’-3’) | Reverse primer (5’-3’) |
| --- | --- | --- |
| *hsa-miR-21* | AGTGTCGTCAGTAGCTTATCAGACT | CTCAACTGGTGTCGTGGAGTC |
| *U6* | CTCGCTTCGGCAGCACA | AACGCTTCACGAATTTGCGT |
| *PTEN* | TGGATTCGACTTAGACTTGACCT | GGTGGGTTATGGTCTTCAAAAGG |
| *PDCD4* | GCAAAAAGGCGACTAAGGAAAAA | TAAGGGCGTCACTCCCACT |
| *Ki-67* | CCACACTGTGTCGTCGTTTG | CCGTGCGCTCATCCATTCA |
| *Bcl-2* | TCCCTCGCTGCACAAATACTC | TTCTGCCCCTGCCAAATCT |
| *Bax* | AACTGGACAGTAACATGGA | CTGGCAAAGTAGAAAAGG |
| *Caspase-3* | CTCCTTCCATCAAATAGAAC | AATTAACAATCATTGCCTCT |
| *Caspase-9* | TAGAAAACCTTACCCCAGT | ATTGTTGATAATGAGGCAGT |
| *BCAR1* | CTGCGTGAGGAGACCTACGA | CAGGAGGAAGCACCCGTTC |
| *KLHDC10* | CTCAACCGCTTCGTGCAAC | CCTAACTGGGTCCCATCGTATTT |
| *ASB8* | CAGAGCAAATACTCTCTCTCCGA | TCTGACACCATACAGGCACAG |
| *FBXO28* | TCCTCAGCTTTATGTCCTACGA | TGGGAGTTGTGCTTTAACTTGT |
| *SLC25A48* | GAGGGAGAGTATGTTCGGCTT | CACCACGGAGTTGTAGACGG |
| *C3orf80* | GAGCACGAGATGCGTGTAGT | TAGGTGGGCAGATACTTGACC |
| *SCN1B* | TCCTGCGCTATGAGAATGAGG | TGGTGTTGTGCTCGTAGTTTTC |
| *β-actin* | GAAG GATT CCTA TGTG GGCG | GATA GCAC AGCC TGGAT AGCA |
